# Expanding biological space coverage enhances the prediction of drug adverse effects in human using *in vitro* activity profiles

**DOI:** 10.1038/s41598-018-22046-w

**Published:** 2018-02-28

**Authors:** Ruili Huang, Menghang Xia, Srilatha Sakamuru, Jinghua Zhao, Caitlin Lynch, Tongan Zhao, Hu Zhu, Christopher P. Austin, Anton Simeonov

**Affiliations:** Division of Pre-clinical Innovation, National Center for Advancing Translational Sciences (NCATS), National Institutes of Health (NIH), Rockville, MD 20850 USA

## Abstract

*In vitro* assay data have recently emerged as a potential alternative to traditional animal toxicity studies to aid in the prediction of adverse effects of chemicals on humans. Here we evaluate the data generated from a battery of quantitative high-throughput screening (qHTS) assays applied to a large and diverse collection of chemicals, including approved drugs, for their capacity in predicting human toxicity. Models were built with animal *in vivo* toxicity data, *in vitro* human cell-based assay data, as well as in combination with chemical structure and/or drug-target information to predict adverse effects observed for drugs in humans. Interestingly, we found that the models built with the human cell-based assay data performed close to those of the models based on animal *in vivo* toxicity data. Furthermore, expanding the biological space coverage of assays by including additional drug-target annotations was shown to significantly improve model performance. We identified a small set of targets, which, when added to the current suite of *in vitro* human cell-based assay data, result in models that greatly outperform those built with the existing animal toxicity data. Assays can be developed for this set of targets to screen compounds for construction of robust models for human toxicity prediction.

## Introduction

Drug safety issues have been the leading cause for attrition during preclinical development as well as in late-stage clinical trials of new drugs^1–4^. After analyzing attrition data for small molecule drug candidates from four large pharmaceutical companies, a study found that preclinical toxicology was the highest cause of attrition during candidate nomination stage, and clinical safety was also a leading cause of attrition in phase I (first) and phase II (second) clinical trials^5^. Even in late stage clinical trials, safety issues remain the leading cause of clinical failure, which account for 25% phase II and 14% phase III failures from 2013 to 2015^6^. Toxicity testing for drugs in development continues to rely heavily on animal models, which are expensive and low throughput with results difficult to translate to humans. To predict the potential toxicological effects of thousands of environmental chemicals, including drugs and drug candidates in early stage of drug development, alternative strategies are required to supplement traditional toxicity testing methods. A number of *in silico* approaches have been developed recently to predict adverse drug reactions using available public datasets of drugs^7–9^. Prediction models were built using chemical structure^10–12^, protein target information^13,14^, phenotypic data^7,15^, or combinations of different data types on drugs, with the application of various machine learning methods. Some of these approaches have shown promising results, yet suffer from a number of limitations. Chemical structure based models rely on structure similarity, thus are often poorly predictive of drugs that are new structure entities. Target information and phenotypic observations are not always available, especially for new drug candidates where early assessment is most critical. Preclinical *in vitro* safety profiling of compounds with biochemical and cellular assays offers an informative and relatively cost-effective approach to complement *in silico* methods^16^. Systematic testing of large chemical libraries to establish a consistent and robust set of *in vitro* activity profiles is challenging but would add tremendous value to improved drug toxicity evaluation^17^.

A major effort addressing this challenge is the U.S. Tox21 (Toxicology in the 21st Century) collaborative effort, which was initiated in 2008 with an emphasis on developing new methodology to evaluate the potential risk of environmental chemicals on human health. The Tox21 program^18–21^ is a collaboration between the National Toxicology Program (NTP) of the National Institute of Environmental Health Sciences (NIEHS), the National Center for Computational Toxicology (NCCT) of the U.S. Environmental Protection Agency (EPA), the National Center for Advancing Translational Sciences (NCATS) of the National Institutes of Health (NIH), and the U.S. Food and Drug Administration (FDA). Tox21 adopted high-throughput screening (HTS) techniques to efficiently test large numbers of chemicals, using the data generated to (1) identify patterns of compound-induced biological responses in order to get insight on toxicity pathways and compound mechanism of toxicity; (2) prioritize compounds for more extensive toxicological evaluation; (3) develop predictive models for biological response in human. The ultimate goal of the Tox21 program is to identify *in vitro* chemical signatures that could act as predictive surrogates for *in vivo* toxicity.

Tox21 has established a library of ~10,000 chemicals for the production phase of the program, including the NCATS Pharmaceutical Collection (NPC), which contains drugs used in the clinic^22^. This library has been screened against 47 cell-based assays in a quantitative high-throughput screening (qHTS) format^23–26^ generating nearly 70 million data points to date. Recently, we have evaluated the utility of these data toward achieving the Tox21 goals^27^. Computational models were built using the *in vitro* assay activity profiles and/or compound structure data to predict *in vivo* toxicity. While useful for generating hypotheses on compound mechanism of toxicity, the assay data based models achieved reasonable but less than ideal performance for most *in vivo* toxicity endpoints, which may be accounted for by species difference (most *in vivo* toxicity endpoints are of animal-model origins while the assays used by Tox21 utilized human cells) and insufficient coverage of the biological space by the assays screened so far, which focused primarily on nuclear receptors^28^ and stress response pathways^29^.

To overcome these limitations and re-evaluate the utility of the *in vitro* human cell-based assay data, here we accessed publicly-available human toxicity data and rebuilt models to predict adverse drug effects on humans. In addition, we tested whether expanded biological space coverage provided by additional *in vitro* data could help improve the predictive performance of the models. As surrogates of *in vitro* data, we incorporated known drug target annotation (DTA) information, such as the protein/gene/pathway target of a drug (e.g. estrogen receptor, TNF signaling pathway) or drug mechanism of action (e.g. dopamine D2-receptor antagonist), on some drugs into these models. Based on the results, we propose a short list of targets/pathways not presently existing within the Tox21 datasets, which can serve as a guide for new assay development and screening toward establishing a robust set of *in vitro* compound activity profiles. Data generated based on these additional targets and pathways may improve the predictive power of the *in vitro* assay data on *in vivo* human toxicity.

## Results

### *In vitro* assay performance and activity

**Figure 1:**
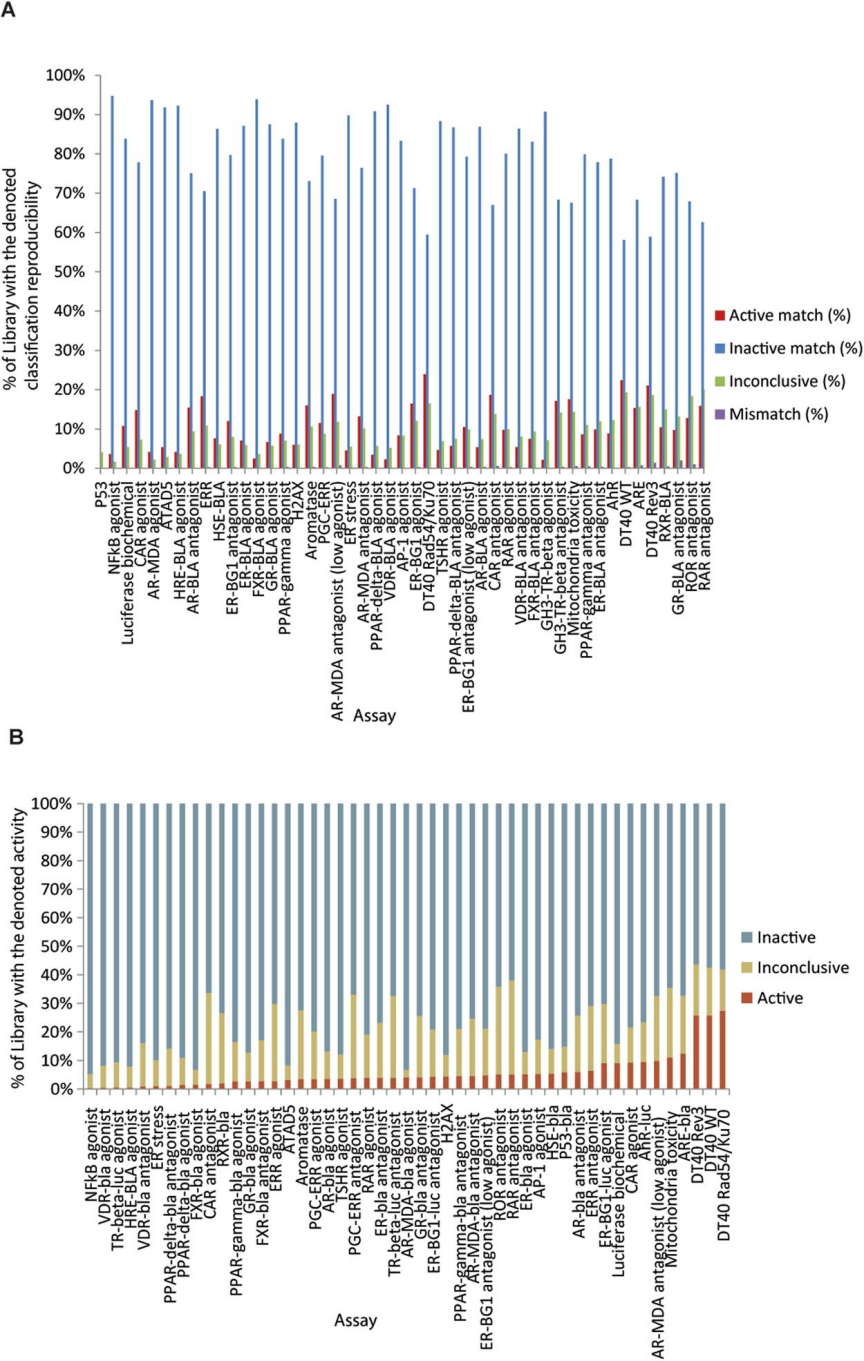
Assay performance (**A**) and activity distribution (**B**) of the Tox21 10 K library screened against 47 assays.

**Figure 2:**
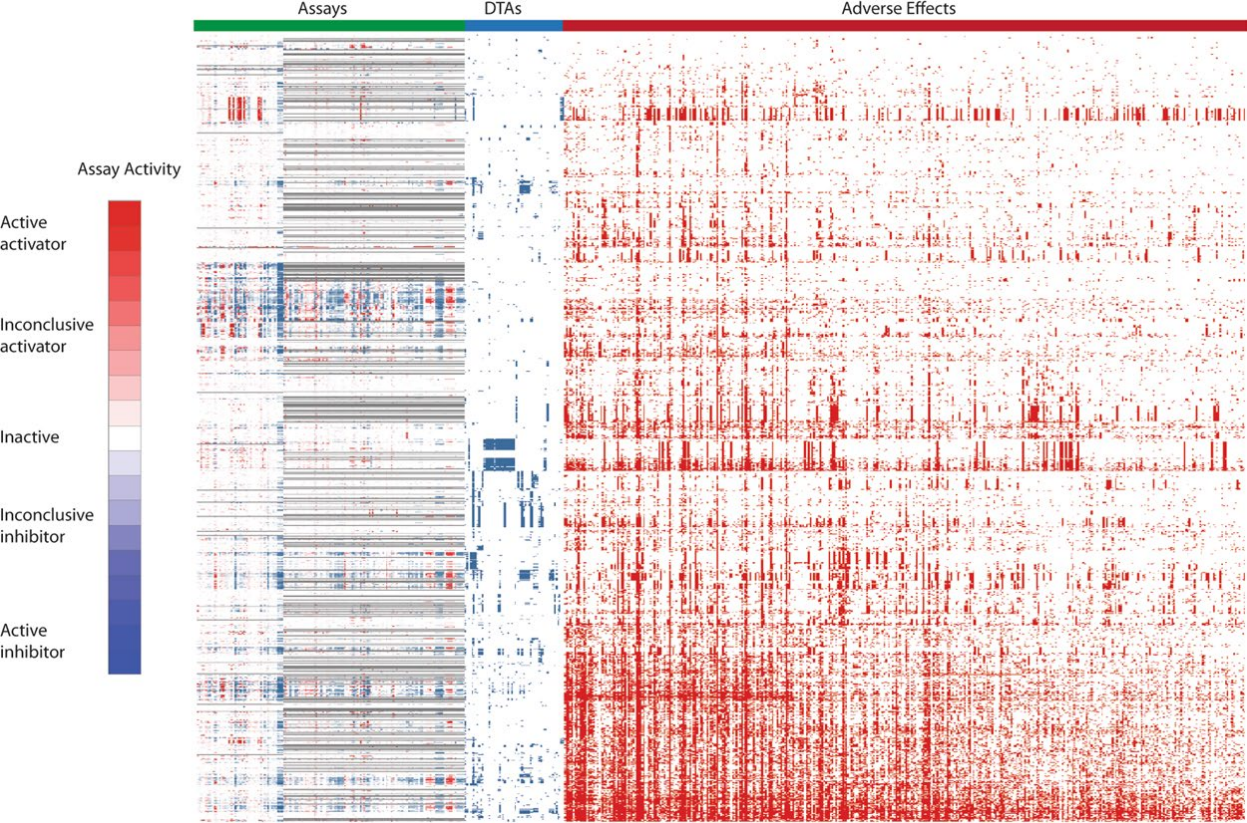
Drug activity and corresponding adverse effect profiles. In the heat map, each row is a drug and each column is an activity measure. Assay activity data are colored by curve rank, such that red indicates activation, blue indicates inhibition, white means inactive, and gray means not tested. A darker shade of red or blue indicates higher confidence in activity. In the DTA profile section, blue means the drug has been reported to have that annotation, and white means no connection has been reported between the drug and the DTA. In the adverse effect profile section, red means the drug has been reported to have that adverse effect, and white means the drug has not been associated with that adverse effect.

As stated in Methods, all data associated with the 47 assays subject to the present analyses are publicly available^30^. Thirty of the 47 assays have been described in detail in our previous study^27^. The performance statistics of all 47 assays in qHTS format are summarized in Table S1. Similar to the 30 previously-described assays, most of the 17 assays screened more recently performed well with signal to background (S/B) ratios ≥ 3-fold, coefficient of variances (CVs) ≤ 10% and Z’ factors ≥ 0.5^31^. The overall performance of an assay is better represented by data reproducibility^25^ in terms of active match, inactive match, inconclusive, and mismatch rates of data generated from the three copies of the compound library with compounds plated in different well locations in each copy (Fig. 1(A)). Forty-two of the 47 assays scored (score = 2 × %active match + %inactive match −%inconclusive − 2 × %mismatch) > 80 (grade A or B) in terms of reproducibility with < 1% mismatches in activity (Table S2). Eleven assays had reproducibility scores between 80 and 90 (grade B) with mismatch rates < 1%. The other five assays scored below 80, but still above 70 with three > 75, with 0.4–2% mismatch rates. The ROR (retinoid-related orphan receptor gamma) and RAR (retinol signaling pathway) antagonist assays were the two lowest scoring assays. For the same sample, the average AC_50_ (50% activity concentration) differences between the three runs were < 2 fold for all the assays (Table S2). The activity distribution of the compounds screened against the 47 assays is shown in Fig. 1(B). The active rates ranged from 0.27% (NFκB agonist mode assay) to 27.4% (DT40 *Rad54/Ku70* mutant assay) with an average active rate of 5.7%. The activity patterns of the NPC compounds across all 47 assays and 156 readouts are better illustrated in Fig. 2 in comparison with their target/mode of action (MOA) annotations and observed adverse effects.

### Predicting human adverse effects using *in vitro* assay and compound structure data

**Figure 3:**
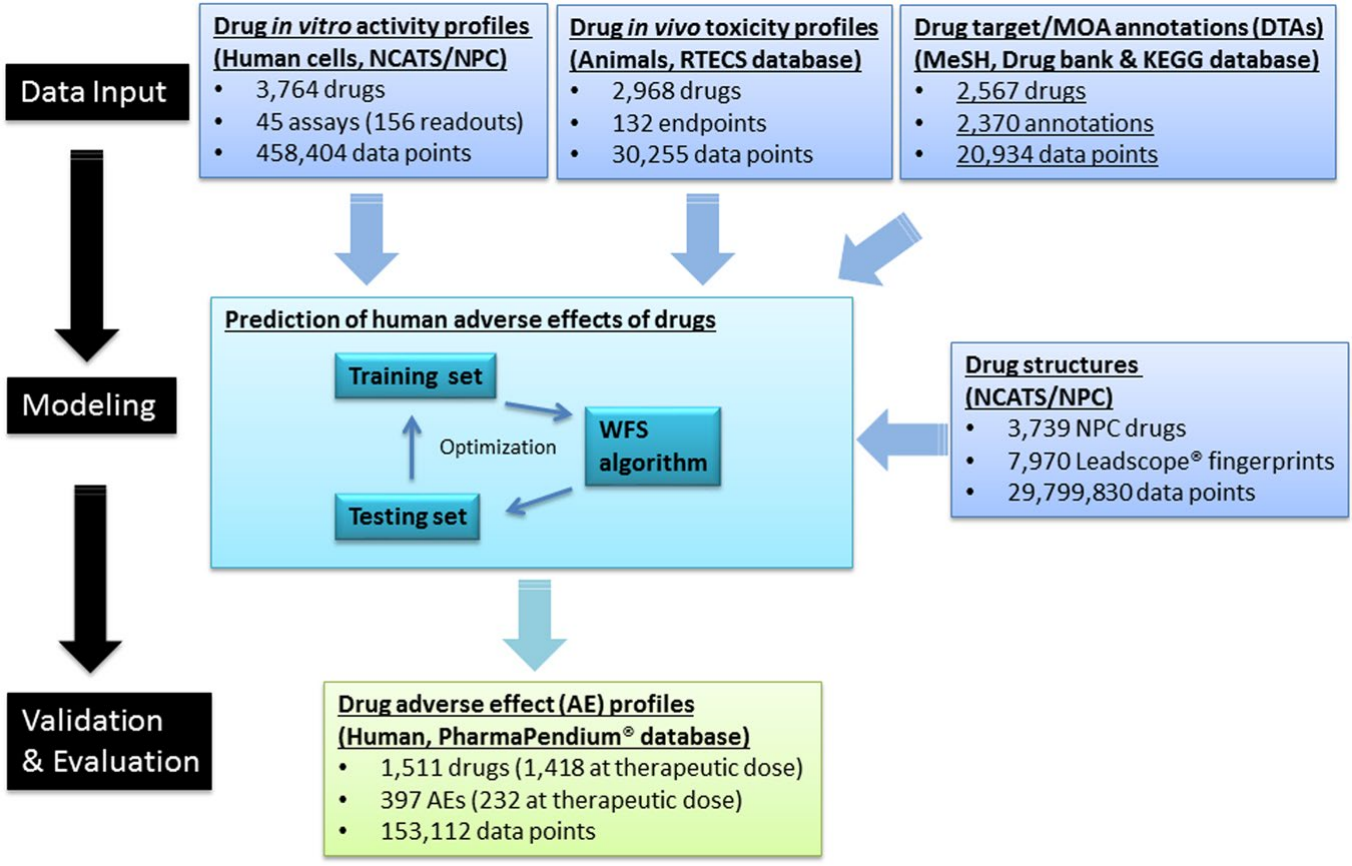
Human ADE predictive modeling process using different datasets.

**Figure 4:**
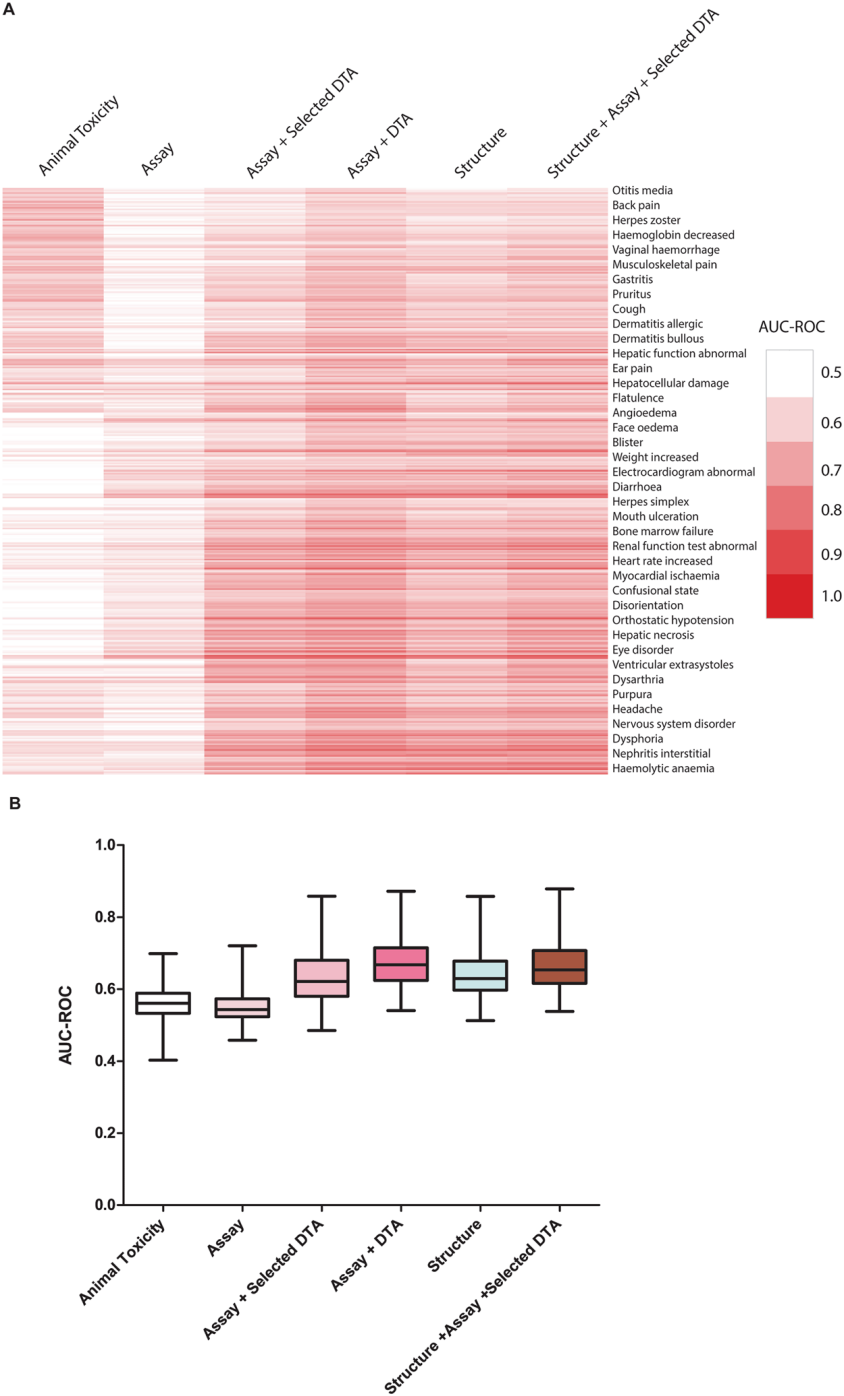
Performance of human ADE prediction models built with different datasets. (**A**) In the heat map, each row is a type of adverse effect, and each column is a different input data type applied in modeling. Heat maps are colored by AUC-ROC value, such that a darker shade of red indicates better model performance and white indicates a random model. (**B**) AUC-ROC value distributions for each input data type applied in modeling.

We evaluated the utility of different types of data in predicting adverse drug effects (ADEs) in human, including *in vitro* assay activity data, compound structure data, and these data in combination with drug-target annotations (DTAs) as illustrated in Fig. 3. The drug activity patterns applied in modeling as well as their ADE profiles are shown in Fig. 2. The performances of these models measured by the average area under the receiver operating characteristic (ROC) curve (AUC-ROC) values are shown in Fig. 4 (see Figure S1 for performances of models built for ADEs at therapeutic dose) and summarized in Table 1. Detailed AUC-ROC results for all the ADE prediction models can be found in Data S1 and S2. Assay activity data by itself showed the lowest, yet statistically significant, predictive power. To test the statistical significance of the model performances, we randomly permutated the activity assignments and rebuilt the ADE models with the randomized activity data. This exercise was repeated 1,000 times and the average AUC-ROC of the models was 0.50 (indicating a completely random model), which is significantly lower than that of the real activity based models (0.55, p < 0.001). The best model built with assay activity data alone is for the ADE “blood prolactin increased” and the ADE “dyskinesia” at therapeutic dose (ADET) with AUC-ROC values of 0.72 and 0.69, respectively. Models built with compound structure data alone achieved much better predictive performance (Table 1). The ADE/ADET prediction models had an average AUC-ROC of 0.64/0.68, with 26 ADEs (28 ADETs) having good predictive models with AUC-ROC values > 0.75. The best ADE and ADET prediction models built with structure data alone are those for “ejaculation disorder” and “nephropathy toxic”, both of which had an AUC-ROC of 0.86. As the activity data used for modeling are from assays that focused only on two areas, nuclear receptor and stress response signaling, the limited biological space coverage is likely a major cause for the low predictive power of the current assay data.

**Table 1:** Performance of human ADE prediction models built with different data sets.

Model	All ADEs (397)			ADEs at therapeutic dose (232)		
	Best AUC-ROC	Mean AUC-ROC	ADEs with AUC-ROC > 0.75	Best AUC-ROC	Mean AUC-ROC	ADEs with AUC-ROC > 0.75
Animal toxicity	0.70	0.56	0	0.69	0.57	0
Assay	0.72	0.55	0	0.69	0.55	0
Structure	0.86	0.64	26	0.86	0.68	28
Assay plus selected DTA	0.86	0.63	22	0.85	0.65	18
Assay plus DTA	0.87	0.67	50	0.87	0.71	66
Structure plus assay plus selected DTA	0.88	0.66	42	0.87	0.70	45

### Predicting human adverse effects using animal data

Animal toxicity tests are traditionally used as surrogates to assess drug toxicity in human. To determine how predictive animal toxicity data are of human toxicity, we also tried to build human ADE prediction models using animal toxicity endpoints from the RTECS database. The resulting models showed predictive performances slightly better than, but not statistically significant (p > 0.05, One-way ANOVA with Bonferroni post hoc test), the models built with assay activity alone with AUC-ROC values averaging 0.56 for the ADE models (compare to 0.55 for the assay data based models) and 0.57 for the ADET models (compare to 0.55 for the assay data based models) (Table 1). Even the best performing models had AUC-ROC values < 0.75 (0.70 for ADE models and 0.69 for ADET models) (Table 1). These results show that even with the limited biological space coverage of the current assay data, the *in vitro* data, using human cell lines, are nearly as predictive of human toxicity as animal data. Interestingly, however, animal toxicity data and *in vitro* assay data seem to be predictive of different types of ADEs as illustrated by Fig. 4. Animal toxicity based models achieved better performance on predicting ADEs such as conduction disorder, pancreatitis, Stevens-Johnson syndrome, respiratory depression, and dermatitis bullous, which had AUC-ROC values > 0.62, than the corresponding *in vitro* assay data based models, the AUC-ROC values of which were < 0.58. In comparison, the assay data based models performed better on predicting ADEs including nephrotic syndrome, extrapyramidal disorder, mania, bacterial infection, and torsade de pointes, with AUC-ROC values > 0.6 and at least larger than those of the corresponding animal toxicity based models by 0.07.

### Expanding biological space coverage improves the predictive power of *in vitro* data on human adverse drug effects

To test if increased biological space coverage could improve the *in vitro* assay activity based models in predicting human ADEs, we used known drug–target annotation (DTA) as additional descriptors and rebuilt the models. When all 2,370 DTAs were combined with *in vitro* assay activity data, the resulting models showed a significant improvement in performance, exceeding the overall performance of the structure based models (p < 0.001, One-way ANOVA with Bonferroni post hoc test) (Table 1). These activity-DTA combined models had average AUC-ROC values of 0.67 and 0.71 for ADEs and ADETs, respectively, both of which are better than those of the structure based models (0.64 for ADEs and 0.68 for ADETs). As the number of DTAs was large, to test if the enhanced model performance was a result of chance or overfitting, we randomly permuted the DTA dataset 1,000 times and reran the activity-DTA combined ADE models. The average AUC-ROC value of the “randomized” models was 0.56, which was comparable to that of the models built with activity data alone, and significantly smaller than that of the real combined models (p < 0.001). Fifty of the ADE models (66 ADET models) showed good predictive power with AUC-ROC > 0.75. The best ADE (papilloedema) and ADET (nephritis interstitial) models had a high AUC-ROC of 0.87. To identify which DTAs contribute the most to improving the prediction performance, each of the DTAs was then evaluated individually. We found a set of 58 DTAs (Table S3) that were predictive of at least one of the human ADEs with AUC-ROC or balanced accuracy (BA) > 0.6. To determine if this set of 58 DTAs could improve the prediction performance of the models, we combined the 58 DTAs with the assay activity data as additional descriptors to build ADE prediction models (Fig. 2). We found that just adding this small set of DTAs significantly improved the predictive performance of the assay activity based models (p < 0.001, One-way ANOVA with Bonferroni post hoc test) (Table 1). The average AUC-ROC increased from 0.55 to 0.63 for the ADE models and 0.55 to 0.65 for the ADET models. Twenty two of the ADE models (18 ADET models) showed good predictive power with AUC-ROC > 0.75 compared to zero for the models built with assay data alone. Moreover, compared with the models built with all DTAs, the performances of the models built with this much smaller selected set of DTAs were only slightly inferior, with the average AUC-ROC decreased from 0.67 to 0.63 for all ADEs and 0.71 to 0.65 for ADETs and the best performing models showing comparably high AUC-ROC values (0.86 for ADEs and 0.85 for ADETs). Finally, we combined the assay activity data with structure data and the selected set of DTAs to build ADE prediction models. These combined models showed performances that were better than those of the assay + DTA (selected) models (mean AUC-ROC increased from 0.63 to 0.66 for ADEs; from 0.65 to 0.70 for ADETs) (p < 0.001, One-way ANOVA with Bonferroni post hoc test), while comparable with those of the assay + DTA (all) models (p > 0.05, One-way ANOVA with Bonferroni post hoc test) (Table 1). Taken together, expanding the biological space coverage of the current assay panel with a small set of DTAs was shown to significantly improve the prediction performance, and the models were further improved by adding compound structure information.

**Figure 5:**
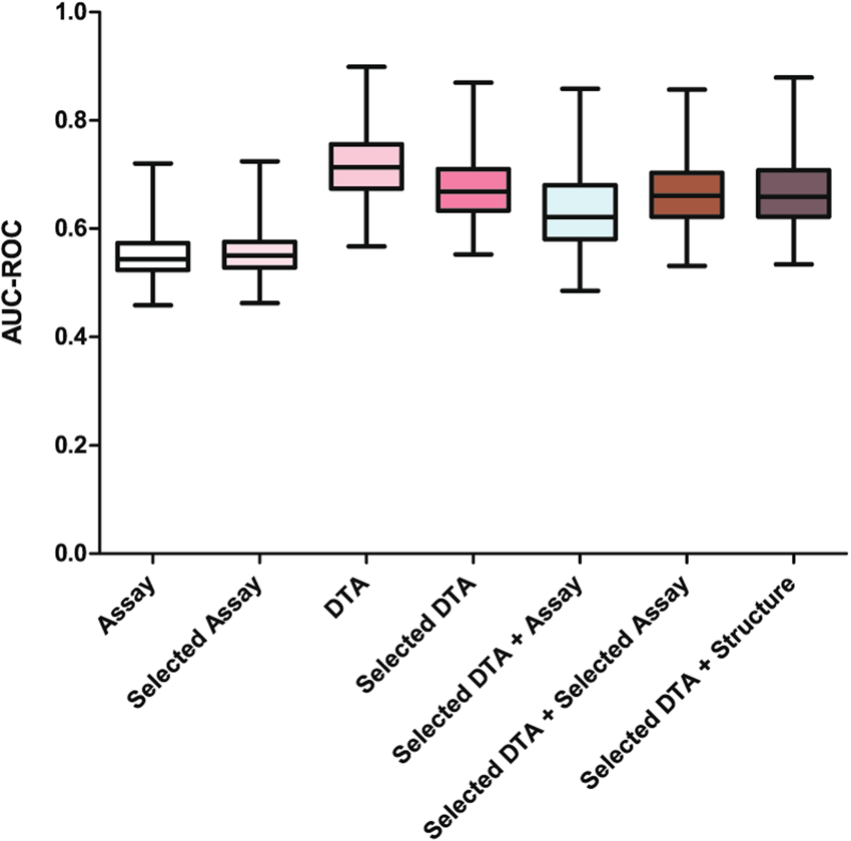
Contribution of DTAs to the performance of human ADE prediction models. The boxplots show the distributions of AUC-ROC values of models built with DTA data alone and in combination with assay or structure data.

To further evaluate the predictive value of the DTAs, we built ADE models with the DTA data alone, using just the 58 selected DTAs as well as all DTA data. These models showed on par or better performance compared to their respective combination models with added assay or structure data (Fig. 5). The models built with all DTA data outperformed all other models with an average AUC-ROC of 0.72 and 114 (28.7%) of the ADEs having AUC-ROC values > 0.75. Adding structure information did not improve model performance – the average AUC-ROC of the models built with the 58 selected DTAs alone was 0.68 while adding structure data decreased the AUC-ROC value slightly to 0.67. Adding assay data did not improve model performance either with an average AUC-ROC value dropping to 0.63 for the selected DTA and assay data combined models. Instead of using all 156 assay readouts, we selected the readouts that were the most predictive with AUC-ROC or balanced accuracy > 0.65 for at least one ADE, and built models with just this selected set of 44 assay readouts. The average AUC-ROC of the assay data alone models improved slightly from 0.55 to 0.56, and that of the models combined with the selected DTAs improved from 0.63 to 0.67, which were on par with the models built in combination with structure data (Fig. 5).

### Comparing prediction performance with traditional classifiers

Given that the goal of this study was to evaluate the value of different data types, *in vitro* assay data in particular, in predicting human *in vivo* toxicity, and not to build optimal models for ADE prediction, we chose to apply one method to model all data types for the results to be consistent and comparable. We chose the WFS method that we have previously shown a robust and flexible method that fits this purpose^32^. In the previous study, we compared WFS with more traditional classifiers such as SVM and Naïve Bayesian for toxicity prediction on a few different datasets^32^. The three methods showed variable but mostly comparable performances depending on the dataset, while WFS outperformed the other two in some cases especially on structurally diverse datasets. To test if the performance of WFS would hold up on ADE modeling, we again compared WFS with three traditional classifiers: support vector machine (SVM), random forest and Naïve Bayes. These three classifiers were applied to build ADE prediction models using the assay data in combination with selected DTA as an example and their performances were compared to those of WFS. WFS (average AUC-ROC = 0.63) outperformed random forest (average AUC-ROC = 0.57) and Naïve Bayes (average AUC-ROC = 0.61), and showed performances similar to SVM (average AUC-ROC = 0.65) (Figure S2). WFS thus appeared to be a method suitable for our purposes and the models could be optimized further when we obtain data from new assays with expanded biological space coverage.

## Discussion

In this study, we tested the applicability of different data types, *in vitro* cell-based assay data in particular, to building predictive models for adverse drug effects in humans by using in-house generated *in vitro* assay data on 1,511 approved drugs, as well as publicly-available human adverse effect data for said drugs. We also conducted the first meta-analysis to compare the performance of animal *in vivo* toxicity data in predicting human adverse outcomes with that of *in vitro* assay data. Animal toxicity data do not seem to have a clear advantage over human cell-based data in predicting human *in vivo* effects. Models built with *in vivo* animal toxicity endpoints showed similarly moderate performance compared to those built with the *in vitro* assay data in predicting adverse drug effects in human. This result again confirms that species differences, as well as data sparsity and lack of consistency, limit the reliability of extrapolating animal *in vivo* toxicity data to human *in vivo* effects.

However, most models built with *in vitro* human cell-based assay data alone did not show good predictive capacity. In comparison, models built with chemical structure information showed better predictive performance for many ADEs. We hypothesized that the low performance of *in vitro* assay data may be due to the limited biological space coverage by the current panel of assays. Therefore, we combined *in vitro* assay data with DTAs collected from the literature (2,370 DTAs) to build new models that showed remarkable improvements in predictive performance. To identify which DTAs contributed the most to the prediction, each of the DTA was then evaluated individually resulting in a set of 58 DTAs that were predictive of at least one of the human ADEs with AUC-ROC or balanced accuracy (BA) > 0.6. Adding the set of 58 DTAs to *in vitro* assay data significantly improved the model performance. Moreover, the assay + DTA models performed better than the structure based models by at least 0.1 in AUC-ROC on a number of ADEs including mania, abnormal behavior, hypercholesterolaemia, ventricular extrasystoles, hiccups and erythema multiforme with AUC-ROC values ranging from 0.70 to 0.83, whereas none of the structure based models outperformed the assay + DTA models by 0.1 in AUC-ROC.

Chemical structure-based prediction of toxicity relies on structure similarity assuming that chemicals sharing similar structure features would exhibit similar biological or toxicological effects. The structure-based models in our study showed good predictive performances for many ADEs. However, structure-based models are not reliable when applied to making predictions on completely new scaffolds not present in the training data. In addition, slightly altered structure features may dramatically change the interaction between a chemical and their targets leading to unexpected toxicity. Models based on *in vitro* assay data circumvent this problem as predictions are based on similarity in activity profiles, with no structure information required, assuming chemicals sharing similar activities would most likely hit the same targets resulting in similar toxicity outcomes. Nevertheless, *in vitro* assay data-based models have their own limitations. Chemicals are often metabolized in the human body whereas most *in vitro* assays do not have metabolic capacity. Therefore, *in vitro* assay data-based models would likely fail in predicting human toxicity caused by these metabolites. One solution for this problem is to introduce metabolic capacity into *in vitro* assays^20,33^. Another solution is to combine *in vitro* assay data with structure data and other available target information. As we have shown in our previous predictive modeling efforts^27^, and further confirmed by the current study, the *combined* models achieved the best performance in predicting human toxicity.

Relative to models built with chemical structure information alone, models built with *in vitro* assay data have the added advantage of identifying the targets and modes of action (MOAs) that contribute the most to each adverse effect, which could provide clues for potential mechanisms leading to the adverse effect. For each adverse effect we evaluated, the most predictive contributors and their corresponding AUC-ROC values are listed in Data S1 and S2, for ADEs and ADETs, respectively. CYP3A4 came out on top as the most predictive contributor for 69 (17%) ADEs and 26 (11%) ADETs. The cytochrome P450 (CYP) isozymes are responsible for the metabolism of approximately two-thirds of known drugs in humans, and CYP3A4 has been suggested to be the most prominent P450 isozyme in drug metabolism and hepatic distribution^34,35^. It is not surprising then that CYP3A4 activity was found to be more predictive in drug toxicity and adverse drug effects. The serotonergic pathway ranked the second among the most predictive contributors, responsible for 23 (6%) ADEs and 26 (11%) ADETs. The serotonin receptors are a group of G protein-coupled receptors (GPCRs) and ligand-gated ion channels found in the central and peripheral nervous systems. The serotonin receptors modulate the release of many neurotransmitters and hormones, and are the target of a variety of drugs such as antidepressants, antipsychotics, anorectics, and antiemetics^36^. Other MOAs identified as the most predictive for at least ten ADEs include “penicillin binding proteins inhibitor” and “anti-bacterial agents”. Of all the *in vitro* assays, the RORγ (retinoic acid-related orphan receptor gamma) antagonist mode assay is the one that contributed to the largest number of ADEs (17, 4%). The specific functions of RORγ have not been fully characterized, but the RORγt isoform has been reported to be a key transcription factor for the differentiation of Th17 cells, which play key pro-inflammatory roles in a variety of autoimmune diseases and in cancer^37,38^. Our findings here suggest a potential role for these DTAs in drug induced toxicity. The FXR (farnesoid X receptor) agonist mode viability assay and the control channel of the GR (glucocorticoid receptor) agonist mode assay were also among the most predictive assays for at least ten ADEs. These are counter screens or background readings that measure the cytotoxic potential of compounds. As a common measure of cytotoxicity, cell viability assays in general turned out to be a major contributor to predicting adverse effects, which were the most predictive contributor for 69 (17%) ADEs and 28 (12%) ADETs.

We have shown that DTA information can significantly improve the predictive performance of the assay data based models. More importantly, data on just a small set of additional DTAs (2% of the entire 2,370 DTA set) that contributed the most to the models can already expand the biological space coverage sufficiently to produce predictive models of human toxicity effect when combined with *in vitro* assay data. While the entire DTA set improved the model performance by 22–28% on average, the selected set of 58 DTAs alone improved the model performance, on average, by 15–18%. That is, 2% of the DTA information could account for ~70% of the improvement in the predictive capacity of the models. It is not surprising that the DTA based models showed the best performance as this data is from the literature and can be considered as validated experimental or assay data, and these DTAs have a good coverage of the drug target space known in the literature. In addition to limited target space coverage, the current assay data used for modeling are primary HTS data without further validation and thus undoubtedly confounded with noise and assay artifacts. These results thereby highlight the importance of data quality and selecting the right assays. Validated DTA data seems to be the best choice for ADE or human *in vivo* toxicity prediction, the DTA based models however, cannot be applied to predict new compounds without such annotations available. It is, therefore, important to generate high quality assay data that have a good coverage of the biological space and have these data validated.

Most of the 58 selected targets/pathways are GPCR targets, such as dopamine receptors, histamine receptors, serotonin receptors, muscarinic cholinergic receptors, etc., which are not covered by the suite of Tox21 qHTS assays screened to date. Nearly 30% of therapeutic agents on the pharmaceutical market target GPCR^39^. Non-specific activity on GPCR targets can lead to undesirable side-effects and other liabilities^40^. Among the 73 drug safety targets that have been associated with adverse drug effects, 67% belong to the GPCR family^10,41^. Also among this selected set of targets are several metabolic pathways, which are not part of the current qHTS assays either, a number of cancer pathways, disease pathways, other signaling pathways and stress response pathways. Two CYP isozymes, CYP3A4 and CYP2D6, which play a major role in drug metabolism, are found within the set of 58 targets as well. The cytochrome P450 (CYP) family contains more than 50 isozymes that are essential for drug metabolism. Six of them metabolize 90 percent of drugs, with CYP3A4 and CYP2D6 being the two most significant^42^. CYPs can be inhibited or induced by many drugs. Inhibition of CYPs may result in drug-drug interactions and cause unanticipated adverse effects. For example, if one drug inhibits the CYP-mediated metabolism of another drug, the second drug may accumulate within the body to a toxic level, especially for drugs with small therapeutic windows^42^. On the other hand, induction of CYPs may result in unexpected accumulation of other drugs or toxins, which need to be bioactivated by CYPs from pro-drugs or pro-toxins within the body, and cause adverse effects^43^. It is not surprising then that the CYPs would contribute to drug adverse effect. Screening assays are available for the CYPs, but they have only been applied against the Tox21 phase I collection of ~3,000 environmental chemicals and a small subset of the NPC library (~800 drugs). Nevertheless, adding these data to the ADE prediction models helped to improve the model performance slightly, yet significantly. The average AUC-ROC values increased from 0.55 to 0.56 for both the ADE and ADET models (t-test: p = 0.02 for the ADE models and p = 0.002 for the ADET models). These targets would thus make a good complement to the current set of qHTS assays such that including them would truly help expand the biological space coverage of these assays. Limited information on known activators/inhibitors of these targets has already shown promise in improving the predictive power of the adverse effect models. Significant added value would be gained from generating data on these targets for the NPC collection and other environmental chemicals and drugs. Many of these targets/pathways, such as the GPCRs, are well-studied disease targets and have probing assays readily available. We proposed a list of assays (Table 2) as a starting point that provide a basic coverage of these 58 targets/pathways. Not all of these currently available assays are high throughput, such as the ELISA-type assays. The commercially available assays and their vendors can be found in Table S4. It would be ideal to develop high throughput assays for the entire set of 58 targets to test the predictive power of the data generated.

**Table 2:** Assays proposed to expand biological space coverage.

High Throughput Assays	High Throughput Assays
Acetylcholinesterase Assays^†^ Bacteria ATP Bioluminescence Assay FLIPR Calcium Assay^49^ Oxytocin Receptor Assay (Calcium Influx)^†^ Cell Transformation *In Vitro* Assay (Colormetric)^50^ Neurotransmitter Transporter Uptake Assay (Fluorescence)^51^ Cyclooxygenase-1 (COX-1) Inhibitor Screening Assay (Fluorometric) Cyclooxygenase-2 (COX-2) Inhibitor Screening Assay (Fluorometric) Penicillin-Binding Proteins Detection Assay (Fluorescence Polarization)^52^ Active Renin ELISA Assay (Fluorometric) Phospho-VEGFR2 (Tyr1175) Cellular Assay (FRET) cGMP Assay (HTRF) Luminescence Cell-Based Platelet Granule Secretion Assay^53^ FLIPR Membrane Potential Assay TRP Channel Assay (Membrane Potential) P450-Glo™ CYP2D6 Assay (Luminescence)^54^ P450-Glo™ CYP3A4 Assay (Luminescence)^54^ MAO-Glo™ Assay	Glucocorticoid Receptor Assay^28^^†^ CRE-bla or CRE-luc Reporter Assay^55^^†^ NFkB-bla or NFkB-luc Reporter Assay^56^^†^ TNF-alpha secretion (HTRF)^57^^†^ Multispan’s GPCR Functional Assay (cAMP/Calcium): ADRA1A, ADRA1B, ADRA1D, ADRA2A, ADRA2B, ADRA2C, ADRB1, ADRB2, ADRB3, CHRM1, CHRM2, CHRM3, CHRM4, CHRM5, DRD1, DRD2, DRD3, DRD4, DRD5, HRH1, HTR1A, HTR1B, HTR1D, HTR1E, HTR2A, HTR2C, HTR6, HTR7
	**Low Throughput Assays**
	Dopamine ELISA Assay Estradiol ELISA Assay Serotonin ELISA Assay Human ALOX5 /5-LOX ELISA Assay Human Gap Junctions PCR Array Rap 1 Western Blot Assay

^†^Assay available from the Tox21 program.

In summary, qHTS *in vitro* assay activity profiles have been evaluated for their predictive capacity of human toxicity manifested as adverse approved-drug effects, alone and in conjunction with structure and/or DTA information. Models built with *in vitro* human assay data alone showed limited predictive power of human effect, possibly due to the limited biological space coverage of the current suite of assays and lack of further validation, but with performance *close* to models built with animal *in vivo* toxicity data. Both chemical structure information and additional DTA annotations significantly improved the predictive performance of the assay data-based models resulting in robust models for many adverse drug effects. Most importantly, just a small set of targets selected to complement the biological space covered by the *in vitro* assays was shown to produce models that performed nearly as well as models built with all DTA information included. This set of targets can serve as guide for assay development in order to generate *in vitro* data that can better predict human toxicity.

## Data and Methods

### *In vitro* assay and structure data

qHTS data generated on the NPC part of the Tox21 10 K collection up to the end of 2015 were used for modeling, including 47 assays with 156 readouts. All data and detailed descriptions of these assays are publicly available through the NCATS website (https://tripod.nih.gov/tox21/assays/) and PubChem^30^. A complete list of assays and readouts can be found in Table S5. Curve rank was used as the measure for compound activity^28^. The detailed process of data normalization, correction, classification of concentration response curves, and activity assignment was described previously^44^. For modeling purposes, compounds with absolute curve rank > 0.5 were set as active (1), and inactive (0) otherwise. Structure fingerprints were generated for compounds in the NPC library using Leadscope® (Leadscope, Inc. Columbus, Ohio, USA) for structure based models.

### *In vivo* toxicity data

Adverse drug effect (ADE) data were extracted from Elsevier’s PharmaPendium® database^45^. PharmaPendium® is a web-based, highly curated database that contains adverse event reports from FDA approval packages and related documents, EMA approval documents, Meylers Side Effects of Drugs, Mosby’s Drug Consult^TM^, and other published toxicity references. For 3,445 drugs 8,629 different ADEs were downloaded. Among those, 7,314 ADEs were observed in humans for 3,316 drugs. These drugs overlap with 1,528 unique compounds in the NPC. After removing ADEs with less than 100 drugs, 397 ADEs associated with 1,511 compounds were kept for modeling. In addition, we extracted the subset of drugs with ADE reported at therapeutic dose, which may be more reliable, for comparison purposes. Again, we removed ADEs with less than 100 drugs. This left us with 1,418 compounds in the NPC with 232 ADEs reported at therapeutic dose for modeling.

Animal toxicity data were retrieved from the Registry of Toxic Effects of Chemical Substances (RTECS) database compiled by Leadscope® (Leadscope, Inc. Columbus, Ohio, USA). This compilation contains 48 acute toxicity endpoints from various species such as rodents, dogs, birds and other mammals on > 10,000 molecules, 2,968 of which overlap with compounds in the NPC library. In addition, RTECS annotates compounds by their toxicity category such as primary irritant, mutagen, reproductive effector, and tumorigen, for a total of 52 endpoints. For acute toxicity endpoints, we followed the Globally Harmonized System (GHS) classification to determine the toxicity cutoff value^46^. Chemicals labeled by the GHS as Categories 1–3 (signal word “DANGER”) were considered toxic. The complete list of *in vivo* toxicity endpoints and their cutoff values for toxicity can be found in Table S6.

### Drug target annotations (DTA)

Medical Subject Headings (MeSH) (http://www.ncbi.nlm.nih.gov/mesh) pharmacological action (PA) terms were used for compound mode of action (MOA) annotations. Gene target annotations for drugs were downloaded from Drug Bank (http://www.drugbank.ca/)^47^. In v3.0 of the database release, 3,228 gene targets were linked to 5,785 drugs. Additional drug target annotations were downloaded from the Kyoto Encyclopedia of Genes and Genomes (KEGG) database (http://www.genome.jp/kegg/) in January of 2016. In this compilation of the database, 3,555 drugs were mapped to 254 human pathways, 4,536 drugs were annotated with 997 gene targets, and 792 drugs were annotated with 33 enzymatic targets. Combining all the drug target and MOA annotations, a total of 2,370 annotations were mapped to 2,567 unique compounds (CAS numbers) in the NPC collection. For modeling purposes, a “1” was assigned to all known drug-target associations and a “0” was assigned if no known association was reported between a drug-target pair.

### Modeling

Models were built for the human ADEs using assay activity (activity based models), compound structure (structure based models), combinations of structure and activity data with or without drug target annotations (DTAs), and animal toxicity endpoints. The Weighted Feature Significance (WFS) method previously developed at NCATS^32^ was applied to construct the models. Briefly, WFS is a two-step scoring algorithm. In the first step, a Fisher’s exact test is used to determine the significance of enrichment for each feature in the drugs with a certain ADE compared to the ones without such ADE reported, and a p-value is calculated for all the features present in the data set. For assay activity data, each assay readout was treated as a feature and the feature value was set to 1 for active compounds and 0 for inactive compounds. For animal *in vivo* toxicity data, each toxicity endpoint was treated as a feature, and the feature value was set to 1 for toxic compounds and 0 for non-toxic compounds. Missing data were omitted from p-value calculations. For structure data, the feature value was set to 1 for drugs containing that structural feature and 0 for drugs that do not have that feature. For DTA data, each DTA was treated as a feature, and the feature value was set to 1 for drugs that reported to have that DTA and 0 for drugs that not known to have the DTA. If a feature is less frequent in the active compound set than the non-active compound set, then its p-value is set to 1. These p-values form what we call a “comprehensive” feature fingerprint, which is then used to score each drug for its potential to cause a certain ADE according to Equation (1), where *p*_*i*_ is the p-value for feature *i*; C is the set of all features present in a drug; M is the set of features encoded in the “comprehensive” feature fingerprint (i.e., features present in at least one drug with that ADE); N is the number of features; and α is the weighting factor, which is set to 1 in all the models described here. A high WFS score indicates a strong potential for ADE.

1 $$WFS=\frac{\sum \mathrm{log}({p}_{i})}{\min (\mathrm{log}({p}_{i}))\times (\alpha {N}_{C-M}+{N}_{M\cap C})}$$

For each model, compounds were randomly split into two groups of approximately equal sizes, one used for training and the other for testing. The randomization was conducted 10 times to generate 10 different training and test sets to evaluate the robustness of the models. Model performance was assessed by calculating the area under the receiver operating characteristic (ROC) curve (AUC-ROC), which is a plot of sensitivity [TP/(TP + FN)] versus (1 − specificity [TN/(TN + FP)])^48^. A perfect model would have an AUC-ROC of 1 and an AUC-ROC of 0.5 indicates a random classifier. The random data split and model training and testing were repeated 10 times, and the average AUC-ROC values were calculated for each model.

Each of the 2,370 DTAs was evaluated for their predictive capacity of the human ADEs using the ROC approach. DTAs (58) found predictive of at least one of the human ADEs at AUC-ROC > 0.6 or with balanced accuracy (BA = (sensitivity + specificity)/2) > 0.6 were selected to compare their impact on model performance with all DTAs.

### Data Availability

The datasets generated during and/or analyzed during the current study are available in PubChem [https://www.ncbi.nlm.nih.gov/pcassay?term = tox21] and from the corresponding author on reasonable request.

## Electronic supplementary material

Supplementary information

Dataset 1
