# Supplementary material for: Expanding biological space coverage enhances the prediction of drug adverse effects in human using in vitro activity profiles
Source: Sci Rep. 2018 Feb 28;8:3783. doi: 10.1038/s41598-018-22046-w (PMC5830476; doi:10.1038/s41598-018-22046-w)
Supplement: Supplementary file 1 — Supplementary information [file 41598_2018_22046_MOESM1_ESM.pdf]

Supplemental Material

Expanding biological space coverage enhances the prediction of drug adverse effects in human using in vitro activity profiles

Ruili Huang, Menghang Xia, Srilatha Sakamuru, Jinghua Zhao, Caitlin Lynch, Tongan Zhao, Hu Zhu, Christopher P. Austin, Anton Simeonov

Table S1. *In vitro* qHTS assay performance summary statistics*.

Assay	S/B†	Z' factor	CV†	Positive Control	Control AC_{50} (μM)†
ATAD5	6.02±0.9	0.73±0.04	13.87±2.31	5-Fluorouridine	2.12±1.66
DT40 *Rad54/Ku70*	40.19±4.12	0.78±0.15	5.63±2.06	Tetra-octyl ammonium bromide	0.42±0.27
DT40 *Rev3*	39.9±6.62	0.79±0.08	6.64±2.63	Tetra-octyl ammonium bromide	0.59±0.48
DT40 WT	39.58±8.88	0.79±0.09	6.33±2.33	Tetra-octyl ammonium bromide	0.44±0.17
H2AX	4.58±0.63	0.54±0.1	6.25±1.93	Camptothecin	0.15±0.03
P53	3.13±0.43	0.61±0.17	6.01±2.27	MitomycinC	1.53±1.30
AP-1 agonist	3.68±0.33	0.6±0.06	18.2±4.81	EGF	1.54E-03±5.47E-04
ARE	2.11±0.3	0.7±0.06	5.36±1.71	β-Naphthoflavone	1.95±0.52
ER stress	2.79±0.23	0.75±0.06	4.77±1.92	17-AAG	0.33±0.07
HRE-BLA agonist	5.75±0.75	0.81±0.04	2.51±0.66	Cobalt(II) chloride hexahydrate	45.9±10.4
HSE-BLA	3.87±0.48	0.46±0.08	3.62±1.17	17-AAG	0.045±0.013
NFkB agonist	9.96±4.24	9.45±77.11	5.54±4.26	TNF alpha	4.80E-05±2.40E-05
Aromatase	6.21±0.79	0.8±0.03	4.68±0.87	Letrozole	6.70E-03±1.61E-03
Luciferase biochemical	63.09±4.34	0.88±0.02	3.27±0.65	PTC124	6.50E-03±2.02E-03

Mitochondria toxicity	5.86±2.99	0.64±0.15	8.25±2.99	FCCP	0.096±0.061
TSHR agonist	5.61±0.76	0.72±0.1	6.55±2.45	TSH	3.39E-04±9.13E-05
AhR	8.23±3.55	0.33±0.17	15.86±14.75	omeprazole	49.5±61.6
AR-BLA agonist	1.85±0.16	0.22±0.1	4.96±1.24	R1881	1.21E-03±1.61E-03
AR-BLA antagonist	2.48±0.28	0.66±0.17	4.24±1.04	Cyproterone acetate	4.68±5.54
AR-MDA agonist	6.63±0.55	0.68±0.06	15.28±2.2	R1881	1.43E-04±4.25E-05
AR-MDA antagonist	17.27±10.03	0.67±0.07	7.81±2.03	Nilutamide	15.3±5.4
AR-MDA antagonist (low agonist)	5.3±0.34	0.71±0.04	6.23±0.98	Nilutamide	0.40±0.06
CAR agonist	4.92±0.35	0.69±0.1	6.04±1.56	CITCO	0.67±0.14
CAR antagonist	3.37±0.35	0.67±0.07	6.71±1.54	PK11195	1.22±0.23
ER-BG1 agonist	2.53±0.28	0.54±0.15	10.29±4.59	Beta-estradiol	2.90E-05±4.20E-04
ER-BG1 antagonist	8.04±0.89	0.77±0.07	5.88±1.8	4-Hydroxy Tamoxifen	0.073±0.009
ER-BG1 antagonist (low agonist)	8.76±1.68	0.76±0.08	5.7±2.43	4-hydroxy Tamoxifen	0.016±0.003
ER-BLA agonist	4.65±0.56	0.53±0.09	3.93±1.58	β-Estradiol	3.32E-04±4.23E-04
ER-BLA antagonist	3.28±0.81	0.41±0.1	11.27±2.58	4-hydroxy Tamoxifen	5.13E-03±1.88E-03
ERR	2.1±0.16	0.62±0.13	2.96±0.88	Genistein	5.59±0.98
FXR-BLA agonist	4±0.68	0.33±0.19	6.98±1.26	Chenodeoxycholic acid	29.8±4.6

FXR-BLA antagonist	4.4±0.86	0.67±0.09	3.47±1.05	Guggulsterone	36.7±7.0
GH3-TR-beta agonist	9.34±2.06	0.63±0.07	12.31±2.95	T3	4.19E-05±4.89E-05
GH3-TR-beta antagonist	4.99±0.94	0.7±0.1	6.1±3.23	N/A	N/A
GR-BLA agonist	2.96±0.15	0.73±0.05	3.33±0.78	Dexamethasone	3.64E-03±9.50E-04
GR-BLA antagonist	1.89±0.12	0.54±0.12	4.84±0.89	Mifeprostone	1.71E-03±7.16E-04
PGC-ERR	3.17±0.2	0.64±0.09	6.17±1.47	Genistein	8.42±2.25
PPAR-delta-BLA agonist	2.51±0.27	0.67±0.06	5.83±1.27	L-165,041	0.036±0.010
PPAR-delta-BLA antagonist	2.2±0.17	0.57±0.06	4.26±0.55	MK886	38.5±5.8
PPAR-gamma agonist	2.41±0.12	0.73±0.04	4.6±2.28	Rosiglitazone	0.012±0.005
PPAR-gamma antagonist	2.05±0.24	0.59±0.28	4.68±1.94	GW9662	2.47E-03±1.39E-03
RAR agonist	5.23±0.96	0.77±0.05	8.98±3.57	Retinol	1.26±2.11
RAR antagonist	4.11±0.19	0.71±0.05	5.59±1.15	ER50891	0.069±0.020
ROR antagonist	18.75±4.38	0.63±0.07	12.99±5.04	TO-901317	4.75±0.85
RXR-BLA	1.91±0.31	0.54±0.22	4.6±2.02	9-cis Retinoic acid	0.020±0.125
VDR-BLA agonist	1.92±0.24	0.46±0.11	4.85±1.04	1alpha, 25-Dihydroxy vitamin D3	3.50E-04±1.71E-04
VDR-BLA antagonist	2.66±0.18	0.55±0.06	6.85±1.15	N/A	N/A

* Data are derived from the positive and negative control wells on each plate and presented as mean ± standard deviation (*n* = 408)

† S/B = Signal to Background, CV = Coefficient of Variance (derived from the negative control wells), AC_{50} = Concentration at 50% activity

Table S2. Assay performances measured by reproducibility of the compound library triplicate runs*.

Assay	Active match (%)	Inactive match (%)	Inconclusive (%)	Mismatch (%)	AC_{50} fold change	Score†	Grade
ATAD5	5.34	91.78	2.84	0.05	1.30	99.51	A
DT40 *Rad54/Ku70*	23.92	59.47	16.50	0.10	1.32	90.61	A
DT40 *Rev3*	21.07	58.89	18.65	1.39	1.49	79.58	C
DT40 WT	22.38	58.07	19.29	0.26	1.40	83.02	B
H2AX	5.96	87.94	6.06	0.04	1.39	93.71	A
P53	11.10	84.86	4.04	0.00	1.29	103.02	A
AP-1 agonist	8.38	83.28	8.29	0.05	1.48	91.65	A
ARE	15.29	68.34	15.65	0.70	1.76	81.85	B
ER stress	4.53	89.78	5.51	0.18	1.50	92.97	A
HRE-BLA agonist	4.16	92.24	3.60	0.00	1.24	96.96	A
HSE-BLA	7.57	86.38	6.05	0.00	1.45	95.46	A
NFkB agonist	3.60	94.76	1.64	0.00	1.23	100.31	A
Aromatase	15.94	73.08	10.66	0.30	1.44	93.66	A
Luciferase biochemical	10.77	83.83	5.40	0.00	1.27	99.97	A
Mitochondria toxicity	17.57	67.52	14.33	0.55	1.53	87.20	B
TSHR agonist	4.64	88.32	6.95	0.08	1.41	90.49	A
AhR	8.86	78.78	12.24	0.10	1.82	84.05	B
AR-BLA agonist	5.36	86.88	7.44	0.30	1.77	89.55	B
AR-BLA antagonist	15.46	75.07	9.38	0.09	1.35	96.44	A
AR-MDA agonist	4.14	93.65	2.20	0.00	1.36	99.74	A
AR-MDA antagonist	13.22	76.44	10.07	0.27	1.48	92.28	A
AR-MDA antagonist (low agonist)	18.93	68.52	11.79	0.72	1.44	93.11	A
CAR agonist	14.78	77.84	7.27	0.10	1.33	99.93	A
CAR antagonist	18.64	66.95	13.83	0.58	1.45	89.24	B

ER-BG1 agonist	16.43	71.22	12.05	0.28	1.52	91.46	A
ER-BG1 antagonist	12.03	79.72	7.96	0.29	1.48	95.25	A
ER-BG1 antagonist (low agonist)	10.49	79.29	9.90	0.31	1.47	89.74	B
ER-BLA agonist	7.01	87.11	5.87	0.01	1.36	95.25	A
ER-BLA antagonist	9.84	77.86	11.95	0.34	1.49	84.90	B
ERR	18.30	70.52	10.89	0.28	1.30	95.66	A
FXR-BLA agonist	2.46	93.87	3.65	0.02	1.53	95.09	A
FXR-BLA antagonist	7.50	83.04	9.35	0.11	1.73	88.48	B
GH3-TR-beta agonist	2.13	90.72	7.15	0.00	1.38	87.84	B
GH3-TR-beta antagonist	17.15	68.35	14.18	0.32	1.39	87.82	B
GR-BLA agonist	6.67	87.49	5.75	0.10	1.37	94.89	A
GR-BLA antagonist	9.73	75.11	13.13	2.01	1.81	77.40	C
PGC-ERR	11.49	79.56	8.69	0.25	1.49	93.35	A
PPAR-delta-BLA agonist	3.46	90.79	5.71	0.04	1.71	91.91	A
PPAR-delta-BLA antagonist	5.67	86.73	7.49	0.10	1.73	90.37	A
PPAR-gamma agonist	8.79	83.87	7.02	0.31	1.61	93.81	A
PPAR-gamma antagonist	8.61	79.88	11.05	0.44	1.90	85.15	B
RAR agonist	9.76	80.06	9.92	0.26	1.63	89.15	B
RAR antagonist	15.89	62.62	20.07	1.40	1.47	71.50	C
ROR antagonist	12.73	67.87	18.38	0.99	1.69	72.95	C
RXR-BLA	10.43	74.17	14.98	0.42	1.69	79.20	C
VDR-BLA agonist	2.25	92.53	5.19	0.03	1.62	91.79	A
VDR-BLA antagonist	5.41	86.41	8.11	0.08	1.53	88.97	B

* Active match is the percentage of compounds that were reproducibly active, inactive match is the percentage of compounds that were reproducibly inactive, and mismatch is the percentage of compounds that showed conflicting activities in the triplicate runs. A compound is assigned inconclusive if its activity in the triplicate runs was not clearly active or inactive to make a

conclusive reproducibility call. Detailed definitions of the reproducibility calls can be found in our previous report[9]. AC_{50} fold change is the average AC_{50} differences in fold of the active compounds in the triplicate runs.

† Score = 2×active match + inactive match – inconclusive - 2×mismatch (Active matches and mismatches are weighed more to distinguish them from inconclusives and to avoid artificially high scores from low active rates.)

Table S3. Drug target annotations (58) found predictive of at least one of the human ADEs at AUC-ROC >0.6 or with balanced accuracy >0.6.

Drug Annotation	Annotation Type
Anti-Bacterial Agents	MOA
Anticonvulsants	MOA
Antihypertensive Agents	MOA
Anti-Inflammatory Agents	MOA
Anti-Inflammatory Agents, Non-Steroidal	MOA
Antipsychotic Agents	MOA
cyclooxygenase-1 (COX-1) inhibitor	MOA
cyclooxygenase-2 (COX-2) inhibitor	MOA
dopamine D2-receptor antagonist	MOA
glucocorticoidreceptor agonist	MOA
H1-receptor antagonist	MOA
penicillin binding proteins inhibitor	MOA
Arachidonic acid metabolism	Pathway
Metabolic pathways	Pathway
Rap1 signaling pathway	Pathway
Calcium signaling pathway	Pathway
cGMP-PKG signaling pathway	Pathway
cAMP signaling pathway	Pathway
NF-kappa B signaling pathway	Pathway
Neuroactive ligand-receptor interaction	Pathway
Adrenergic signaling in cardiomyocytes	Pathway
VEGF signaling pathway	Pathway
Gap junction	Pathway
Platelet activation	Pathway
TNF signaling pathway	Pathway
Retrograde endocannabinoid signaling	Pathway
Cholinergic synapse	Pathway

Serotonergic synapse	Pathway
Dopaminergic synapse	Pathway
Taste transduction	Pathway
Inflammatory mediator regulation of TRP channels	Pathway
Ovarian steroidogenesis	Pathway
Oxytocin signaling pathway	Pathway
Regulation of lipolysis in adipocytes	Pathway
Renin secretion	Pathway
Salivary secretion	Pathway
Parkinson's disease	Pathway
Cocaine addiction	Pathway
Morphine addiction	Pathway
Nicotine addiction	Pathway
Alcoholism	Pathway
Leishmaniasis	Pathway
Pathways in cancer	Pathway
Chemical carcinogenesis	Pathway
MicroRNAs in cancer	Pathway
Small cell lung cancer	Pathway
Dilated cardiomyopathy	Pathway
CHRM1	Target
CHRM2	Target
CHRM3	Target
CYP2D6	Target
CYP3A4	Target
DRD2	Target
HRH1	Target
HTR2A	Target
PTGS1	Target
PTGS2	Target

SLC6A4	Target

Table S4. Commercial vendors for assays proposed to expand pathway coverage.

Assay	Vendor URL
Acetylcholine Assays	http://www.cellbiolabs.com/acetylcholine-assay-kit?gclid=CjwKEAiA9s_BBRCL3ZKWsfblgS8SJACbST7DGiEcxjLMUiKcexN5jWrZKe_F0rvYfRg51KG727y0ZxoCmdPw_wcB
Cell Transformation In Vitro Assay (Colormetric)	http://www.biovision.com/cell-transformation-assay-kit-colorimetric-12185.html?gclid=CLaN886crtACFUlMDQod0PUCmQ
Neurotransmitter Transporter Uptake Assay (Fluorescence)	https://www.moleculardevices.com/products/assay-kits/neurotransmitter-transporter-uptake-assay-kit
Cyclooxygenase-1 (COX-1) Inhibitor Screening Assay (Fluorometric)	http://www.biovision.com/cyclooxygenase-1-cox-1-inhibitor-screening-kit-fluorometric-9853.html
Cyclooxygenase-2 (COX-2) Inhibitor Screening Assay (Fluorometric)	http://www.biovision.com/cox-2-inhibitor-screening-kit-fluorometric-9891.html
Active Renin ELISA Assay (Fluorometric)	http://www.cisbio.com/other/diagnostics/products/endocrinology-hypertension/active-renin-elisa-assay-kit
Phospho-VEGFR2 (Tyr1175) Cellular Assay (FRET)	http://www.cisbio.com/china/drug-discovery/phospho-vegfr2-tyr1175-cellular-assay-kit
cGMP Assay (HTRF)	http://www.cisbio.com/usa/drug-discovery/cgmp-assay
Luminescence Cell-Based Platelet Granule Secretion Assay	https://bard.nih.gov/BARD/assayDefinition/show/4013
FLIPR Membrane Potential Assay	https://www.moleculardevices.com/products/assay-kits/flipr-membrane-potential-assay-kits
TRP Channel Assay (Membrane Potential)	http://www.sbdrugdiscovery.com/trp-channels.asp
P450-Glo™ CYP2D6 Assay (Luminescence)	https://www.promega.com/-/media/files/resources/cell-notes/cn014/p450-glo-cyp2c19-and-cyp2d6-assays-and-screening-systems.pdf?la=en

P450-Glo™ CYP3A4 Assay (Luminescence)	https://www.promega.com/-/media/files/resources/cell-notes/cn023/cyp3a4-p450-glo-assays-with-luciferin-ipa.pdf?la=en
MAO-Glo™ Assay	https://www.promega.com/products/cell-health-and-metabolism/adme-assays/mao_glo-assay-systems/
Multispan's GPCR Functional Assay (cAMP/Calcium)	http://www.multispaninc.com/
Dopamine ELISA Assay	http://www.eaglebio.com/dopamine-elisa-assay-kit/?gclid=CKS2t_CfqdACFUhMDQodQp0N8A
Estradiol ELISA Assay	https://www.thermofisher.com/order/catalog/product/KAQ0621
	http://www.eaglebio.com/estradiol-elisa-assay-kit/?gclid=CKDBtt6Vq9ACFU5XDQodBm4Fsg
Serotonin ELISA Assay	http://www.abcam.com/products?selected.classification=ELISA%2C+Matched+Antibody+Pairs+and+Multiplex+Immunoassays--ELISA+Kits+and+Reagents&selected.targetName=Serotonin&gclid=CjwKEAiA9s_BBRCL3ZKWsfblgS8SJACbST7DytA89c_xHWqkwmY7_6XGuVylpj7IcVdpuwe_HrM0_RoCvlTw_wcB
Human ALOX5 / 5-LOX ELISA Assay	https://www.lsbio.com/elisakits/human-alox5-5-lox-elisa-kit-sandwich-elisa-ls-f20873/20873?trid=247
Human Gap Junctions PCR Array	http://www.sabiosciences.com/rt_pcr_product/HTML/PAHS-144Z.html
Bacteria ATP Bioluminescence Assay	https://www.promega.com/search-results/?q=ATP bioluminescence assay in bacteria
FLIPR Calcium Assay	https://www.moleculardevices.com/products/assay-kits/flipr-calcium-assay-kits
Rap 1 Western Blot Assay	https://www.thermofisher.com/antibody/product/RAP1-Antibody-Polyclonal/PA5-27588

Table S5. Tox21 *in vitro* assays and readouts.

Assay Code	Assay Readout
tox21-ahr-p1	AhR
tox21-ahr-p1	AhR viability*
tox21-ap1-agonist-p1	AP-1 agonist ch1
tox21-ap1-agonist-p1	AP-1 agonist ch2
tox21-ap1-agonist-p1	AP-1 agonist ratio*
tox21-ap1-agonist-p1	AP-1 agonist viability
tox21-ar-bla-agonist-p1	AR-BLA agonist ch1
tox21-ar-bla-agonist-p1	AR-BLA agonist ch2*
tox21-ar-bla-agonist-p1	AR-BLA agonist ratio*
tox21-ar-bla-antagonist-p1	AR-BLA antagonist ch1
tox21-ar-bla-antagonist-p1	AR-BLA antagonist ch2*
tox21-ar-bla-antagonist-p1	AR-BLA antagonist ratio*
tox21-ar-bla-antagonist-p1	AR-BLA antagonist viability
tox21-are-bla-p1	ARE ch1
tox21-are-bla-p1	ARE ch2
tox21-are-bla-p1	ARE ratio
tox21-are-bla-p1	ARE viability
tox21-ar-mda-kb2-luc-agonist-p1	AR-MDA agonist*
tox21-ar-mda-kb2-luc-antagonist-p1	AR-MDA antagonist
tox21-ar-mda-kb2-luc-antagonist-p2	AR-MDA antagonist (low agonist)*
tox21-ar-mda-kb2-luc-antagonist-p1	AR-MDA antagonist viability
tox21-ar-mda-kb2-luc-antagonist-p2	AR-MDA antagonist viability (low agonist)
tox21-aromatase-p1	Aromatase
tox21-aromatase-p1	Aromatase viability
tox21-elg1-luc-agonist-p1	ATAD5 agonist
tox21-elg1-luc-agonist-p1	ATAD5 viability
tox21-car-agonist-p1	CAR agonist ratio
tox21-car-agonist-p1	CAR agonist viability*

tox21-car-antagonist-p1	CAR antagonist ratio*
tox21-car-antagonist-p1	CAR antagonist viability*
tox21-dt40-p1	DT40 Rad54/Ku70 *
tox21-dt40-p1	DT40 Rev3 *
tox21-dt40-p1	DT40 WT *
tox21-esre-bla-p1	ER stress ch1
tox21-esre-bla-p1	ER stress ch2
tox21-esre-bla-p1	ER stress ratio
tox21-esre-bla-p1	ER stress viability
tox21-er-luc-bg1-4e2-agonist-p2	ER-BG1 agonist
tox21-er-luc-bg1-4e2-antagonist-p1	ER-BG1 antagonist
tox21-er-luc-bg1-4e2-antagonist-p2	ER-BG1 antagonist (low agonist)
tox21-er-luc-bg1-4e2-antagonist-p1	ER-BG1 antagonist viability
tox21-er-luc-bg1-4e2-antagonist-p2	ER-BG1 antagonist viability (low agonist)
tox21-er-bla-agonist-p2	ER-BLA agonist ch1*
tox21-er-bla-agonist-p2	ER-BLA agonist ch2*
tox21-er-bla-agonist-p2	ER-BLA agonist ratio
tox21-er-bla-antagonist-p1	ER-BLA antagonist ch1
tox21-er-bla-antagonist-p1	ER-BLA antagonist ch2
tox21-er-bla-antagonist-p1	ER-BLA antagonist ratio*
tox21-er-bla-antagonist-p1	ER-BLA antagonist viability
tox21-err-p1	ERR*
tox21-err-p1	ERR viability*
tox21-fxr-bla-agonist-p2	FXR-BLA agonist ch1
tox21-fxr-bla-agonist-p2	FXR-BLA agonist ch2
tox21-fxr-bla-agonist-p2	FXR-BLA agonist ratio
tox21-fxr-bla-agonist-p2	FXR-BLA agonist viability*
tox21-fxr-bla-antagonist-p1	FXR-BLA antagonist ch1
tox21-fxr-bla-antagonist-p1	FXR-BLA antagonist ch2*
tox21-fxr-bla-antagonist-p1	FXR-BLA antagonist ratio*
tox21-fxr-bla-antagonist-p1	FXR-BLA antagonist viability*

tox21-gr-hela-bla-agonist-p1	GR-BLA agonist ch1*
tox21-gr-hela-bla-agonist-p1	GR-BLA agonist ch2
tox21-gr-hela-bla-agonist-p1	GR-BLA agonist ratio*
tox21-gr-hela-bla-antagonist-p1	GR-BLA antagonist ch1
tox21-gr-hela-bla-antagonist-p1	GR-BLA antagonist ch2
tox21-gr-hela-bla-antagonist-p1	GR-BLA antagonist ratio
tox21-gr-hela-bla-antagonist-p1	GR-BLA antagonist viability*
tox21-h2ax-cho-p2	H2AX ch1*
tox21-h2ax-cho-p2	H2AX ch2
tox21-h2ax-cho-p2	H2AX ratio*
tox21-h2ax-cho-p2	H2AX viability
tox21-hre-bla-agonist-p1	HRE-BLA agonist ch1
tox21-hre-bla-agonist-p1	HRE-BLA agonist ch2
tox21-hre-bla-agonist-p1	HRE-BLA agonist ratio
tox21-hre-bla-agonist-p1	HRE-BLA agonist viability*
tox21-hse-bla-p1	HSE-BLA ch1
tox21-hse-bla-p1	HSE-BLA ch2
tox21-hse-bla-p1	HSE-BLA ratio
tox21-hse-bla-p1	HSE-BLA viability
tox21-luc-biochem-p1	Luciferase biochemical
tox21-mitotox-p1	Mitochondria toxicity FITC
tox21-mitotox-p1	Mitochondria toxicity ratio
tox21-mitotox-p1	Mitochondria toxicity rhodamine
tox21-mitotox-p1	Mitochondria toxicity viability
tox21-nfkb-bla-agonist-p1	NFkB agonist ch1
tox21-nfkb-bla-agonist-p1	NFkB agonist ch2
tox21-nfkb-bla-agonist-p1	NFkB agonist ratio
tox21-nfkb-bla-agonist-p1	NFkB agonist viability
tox21-p53-bla-p1	P53 ch1
tox21-p53-bla-p1	P53 ch2
tox21-p53-bla-p1	P53 ratio*

tox21-p53-bla-p1	P53 viability
tox21-pgc-err-p1	PGC-ERR
tox21-pgc-err-p1	PGC-ERR viability*
tox21-ppard-bla-agonist-p1	PPAR-delta-BLA agonist ch1
tox21-ppard-bla-agonist-p1	PPAR-delta-BLA agonist ch2
tox21-ppard-bla-agonist-p1	PPAR-delta-BLA agonist ratio
tox21-ppard-bla-agonist-p1	PPAR-delta-BLA agonist viability*
tox21-ppard-bla-antagonist-p1	PPAR-delta-BLA antagonist ch1
tox21-ppard-bla-antagonist-p1	PPAR-delta-BLA antagonist ch2
tox21-ppard-bla-antagonist-p1	PPAR-delta-BLA antagonist ratio
tox21-ppard-bla-antagonist-p1	PPAR-delta-BLA antagonist viability*
tox21-pparg-bla-agonist-p1	PPAR-gamma agonist ch1
tox21-pparg-bla-agonist-p1	PPAR-gamma agonist ch2
tox21-pparg-bla-agonist-p1	PPAR-gamma agonist ratio
tox21-pparg-bla-antagonist-p1	PPAR-gamma antagonist ch1
tox21-pparg-bla-antagonist-p1	PPAR-gamma antagonist ch2
tox21-pparg-bla-antagonist-p1	PPAR-gamma antagonist ratio
tox21-pparg-bla-antagonist-p1	PPAR-gamma antagonist viability
tox21-rar-agonist-p1	RAR agonist
tox21-rar-antagonist-p2	RAR antagonist*
tox21-rar-viability-p1	RAR viability
tox21-rar-viability-p2	RAR viability
tox21-rt-viability-hek293-p1	Real time viability HEK293 flor 0 hr
tox21-rt-viability-hek293-p1	Real time viability HEK293 flor 16 hr
tox21-rt-viability-hek293-p1	Real time viability HEK293 flor 24 hr
tox21-rt-viability-hek293-p1	Real time viability HEK293 flor 32 hr
tox21-rt-viability-hek293-p1	Real time viability HEK293 flor 40 hr
tox21-rt-viability-hek293-p1	Real time viability HEK293 flor 8 hr
tox21-rt-viability-hek293-p1	Real time viability HEK293 glo 0 hr
tox21-rt-viability-hek293-p1	Real time viability HEK293 glo 16 hr*
tox21-rt-viability-hek293-p1	Real time viability HEK293 glo 24 hr*

tox21-rt-viability-hek293-p1	Real time viability HEK293 glo 32 hr*
tox21-rt-viability-hek293-p1	Real time viability HEK293 glo 40 hr*
tox21-rt-viability-hek293-p1	Real time viability HEK293 glo 8 hr
tox21-rt-viability-hepg2-p1	Real time viability HepG2 flor 0 hr
tox21-rt-viability-hepg2-p1	Real time viability HepG2 flor 16 hr
tox21-rt-viability-hepg2-p1	Real time viability HepG2 flor 24 hr
tox21-rt-viability-hepg2-p1	Real time viability HepG2 flor 32 hr
tox21-rt-viability-hepg2-p1	Real time viability HepG2 flor 40 hr
tox21-rt-viability-hepg2-p1	Real time viability HepG2 flor 8 hr
tox21-rt-viability-hepg2-p1	Real time viability HepG2 glo 0 hr
tox21-rt-viability-hepg2-p1	Real time viability HepG2 glo 16 hr
tox21-rt-viability-hepg2-p1	Real time viability HepG2 glo 24 hr
tox21-rt-viability-hepg2-p1	Real time viability HepG2 glo 32 hr
tox21-rt-viability-hepg2-p1	Real time viability HepG2 glo 40 hr
tox21-rt-viability-hepg2-p1	Real time viability HepG2 glo 8 hr
tox21-ror-cho-antagonist-p1	ROR antagonist*
tox21-ror-cho-viability-p1	ROR viability
tox21-rxr-bla-agonist-p1	RXR-BLA ch1
tox21-rxr-bla-agonist-p1	RXR-BLA ch2
tox21-rxr-bla-agonist-p1	RXR-BLA ratio
tox21-rxr-bla-agonist-p1	RXR-BLA viability*
tox21-gh3-tre-agonist-p1	TR-beta agonist
tox21-gh3-tre-antagonist-p1	TR-beta antagonist*
tox21-gh3-tre-antagonist-p1	TR-beta antagonist viability*
tox21-tshr-agonist-p1	TSHR agonist ch1
tox21-tshr-agonist-p1	TSHR agonist ch2
tox21-tshr-agonist-p1	TSHR agonist ratio
tox21-vdr-bla-agonist-p1	VDR-BLA agonist ch1
tox21-vdr-bla-agonist-p1	VDR-BLA agonist ch2
tox21-vdr-bla-agonist-p1	VDR-BLA agonist ratio
tox21-vdr-bla-agonist-p1	VDR-BLA agonist viability

tox21-vdr-bla-antagonist-p1	VDR-BLA antagonist ch1
tox21-vdr-bla-antagonist-p1	VDR-BLA antagonist ch2
tox21-vdr-bla-antagonist-p1	VDR-BLA antagonist ratio*
tox21-vdr-bla-antagonist-p1	VDR-BLA antagonist viability*

*Assay readouts that were predictive of at least one ADE with AUC-ROC or balanced accuracy >0.65.

Table S6. Animal in vivo toxicity endpoints and cutoff values for toxicity.

In Vivo Toxicity Endpoint	Toxicity Cutoff
Acute toxicity LC50 Mammal Inhalation micro-g/mL	1
Acute toxicity LC50 Mouse Inhalation micro-g/mL	1
Acute toxicity LC50 Mouse Inhalation ppm	2500
Acute toxicity LC50 Rat Inhalation micro-g/mL	1
Acute toxicity LC50 Rat Inhalation ppm	2500
Acute toxicity LD50 Bird Oral mg/kg	300
Acute toxicity LD50 Cat Injection - intravenous mg/kg	25
Acute toxicity LD50 Cat Oral mg/kg	300
Acute toxicity LD50 Chicken Oral mg/kg	300
Acute toxicity LD50 Dog Injection - intravenous mg/kg	25
Acute toxicity LD50 Dog Oral mg/kg	300
Acute toxicity LD50 Duck Oral mg/kg	300
Acute toxicity LD50 Guinea pig Injection - intravenous mg/kg	25
Acute toxicity LD50 Guinea pig Injection - subcutaneous mg/kg	148
Acute toxicity LD50 Guinea pig Intraperitoneal mg/kg	96
Acute toxicity LD50 Guinea pig Oral mg/kg	300
Acute toxicity LD50 Guinea pig Skin mg/kg	1000
Acute toxicity LD50 Hamster Oral mg/kg	300
Acute toxicity LD50 Mammal Injection - intravenous mg/kg	25
Acute toxicity LD50 Mammal Injection - subcutaneous mg/kg	148
Acute toxicity LD50 Mammal Intraperitoneal mg/kg	96
Acute toxicity LD50 Mammal mg/kg	133.5
Acute toxicity LD50 Mammal Oral mg/kg	300
Acute toxicity LD50 Mouse Injection - intravenous mg/kg	25
Acute toxicity LD50 Mouse Injection - subcutaneous mg/kg	148
Acute toxicity LD50 Mouse Intracerebral mg/kg	4
Acute toxicity LD50 Mouse Intramuscular mg/kg	134
Acute toxicity LD50 Mouse Intraperitoneal mg/kg	96

Acute toxicity LD50 Mouse mg/kg	133.5
Acute toxicity LD50 Mouse Oral mg/kg	300
Acute toxicity LD50 Mouse Parenteral mg/kg	32
Acute toxicity LD50 Mouse Skin mg/kg	1000
Acute toxicity LD50 Quail Oral mg/kg	300
Acute toxicity LD50 Rabbit Injection - intravenous mg/kg	25
Acute toxicity LD50 Rabbit Injection - subcutaneous mg/kg	148
Acute toxicity LD50 Rabbit Intraperitoneal mg/kg	96
Acute toxicity LD50 Rabbit Oral mg/kg	300
Acute toxicity LD50 Rabbit Skin mg/kg	1000
Acute toxicity LD50 Rabbit Skin mL/kg	0.8425
Acute toxicity LD50 Rat Injection - intravenous mg/kg	25
Acute toxicity LD50 Rat Injection - subcutaneous mg/kg	148
Acute toxicity LD50 Rat Intramuscular mg/kg	134
Acute toxicity LD50 Rat Intraperitoneal mg/kg	96
Acute toxicity LD50 Rat mg/kg	133.5
Acute toxicity LD50 Rat Oral mg/kg	300
Acute toxicity LD50 Rat Oral mL/kg	300
Acute toxicity LD50 Rat Parenteral mg/kg	32
Acute toxicity LD50 Rat Skin mg/kg	1000
Primary Irritant	As classified
Mutagen	As classified
Reproductive Effector	As classified
Tumorigen	As classified

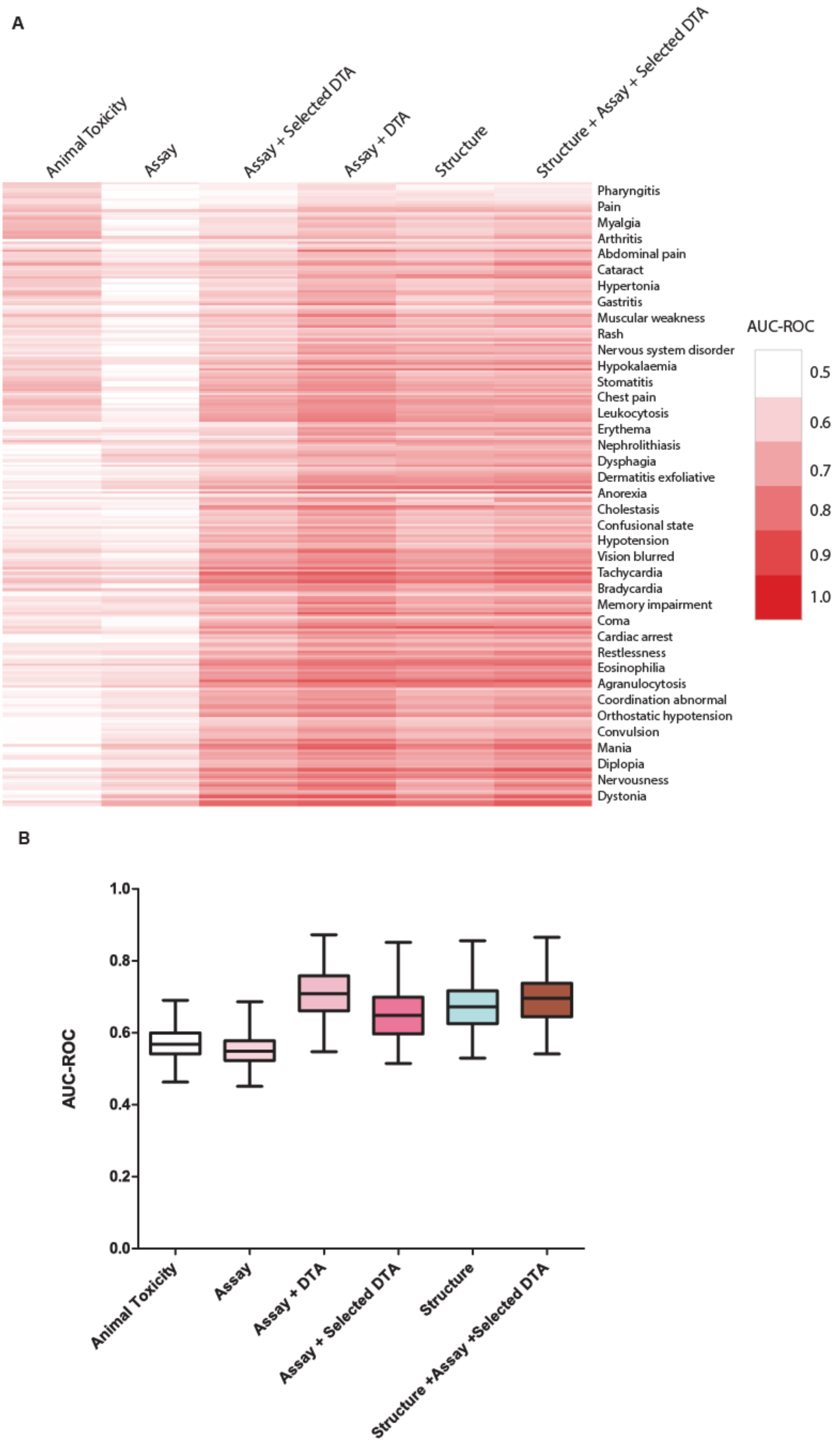
A
Animal Toxicity
Assay
Assay + Selected DTA
Assay + DTA
Structure
Structure + Assay + Selected DTA
Pharyngitis
Pain
Myalgia
Arthritis
Abdominal pain
Cataract
Hypertonia
Gastritis
Muscular weakness
Rash
Nervous system disorder
Hypokalaemia
Stomatitis
Chest pain
Leukocytosis
Erythema
Nephrolithiasis
Dysphagia
Dermatitis exfoliative
Anorexia
Cholestasis
Confusional state
Hypotension
Vision blurred
Tachycardia
Bradycardia
Memory impairment
Coma
Cardiac arrest
Restlessness
Eosinophilia
Agranulocytosis
Coordination abnormal
Orthostatic hypotension
Convulsion
Mania
Diplopia
Nervousness
Dystonia
AUC-ROC
0.5
0.6
0.7
0.8
0.9
1.0
B
AUC-ROC
1.0
0.8
0.6
0.4
0.2
0.0
Animal Toxicity
Assay
Assay + DTA
Assay + Selected DTA
Structure
Structure +Assay +Selected DTA

Figure S1. Performance of human adverse drug effect (ADE) prediction models for ADEs observed at therapeutic dose. (a) In the heat map, each row is a type of adverse effect, and each column is a different input data type applied in modeling. The heat map is colored by AUC-ROC value, such that a darker shade of red indicates better model performance and white indicates a random model. (b) AUC-ROC value distributions for each input data type applied in modeling.

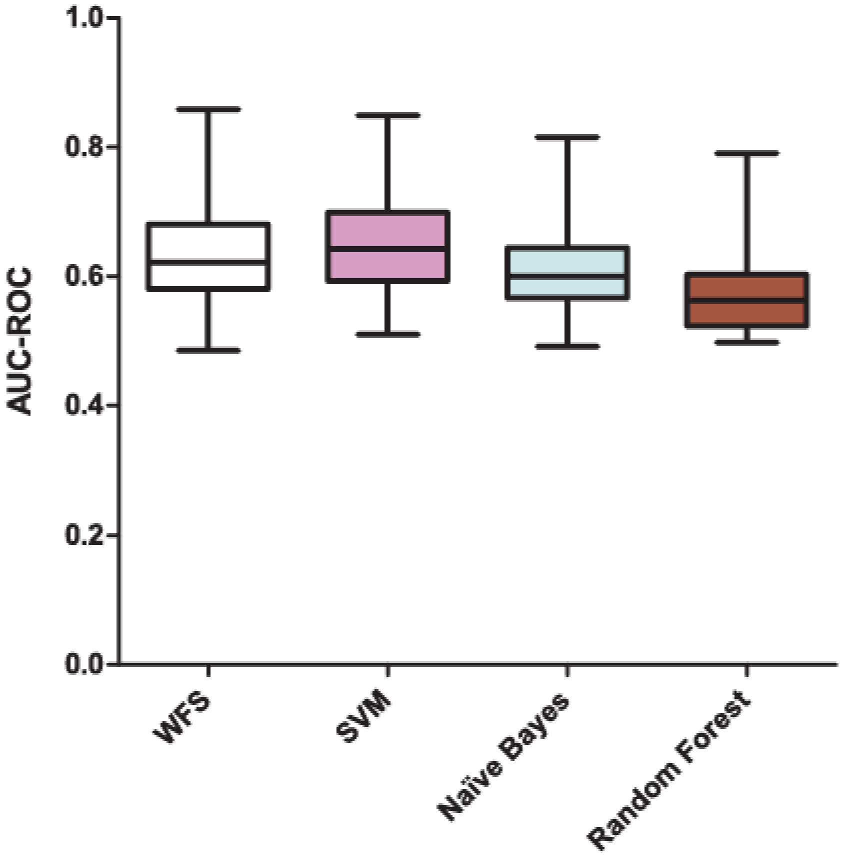

Figure S2. Performance of human adverse drug effect (ADE) prediction models based on *in vitro* assay activity combined with selected DTA using the WFS algorithm compared with traditional classifiers: SVM, Naïve Bayes and Random Forest. The boxplots show the distributions of model AUC-ROC values.
